# Semi-Automated Live Tracking of Microglial Activation in CX3CR1^GFP^ Mice During Experimental Autoimmune Encephalomyelitis by Confocal Scanning Laser Ophthalmoscopy

**DOI:** 10.3389/fimmu.2021.761776

**Published:** 2021-10-21

**Authors:** Moritz J. Frenger, Christina Hecker, Mustafa Sindi, Andrea Issberner, Hans-Peter Hartung, Sven G. Meuth, Michael Dietrich, Philipp Albrecht

**Affiliations:** ^1^ Department of Neurology, Heinrich-Heine University Düsseldorf, Medical Faculty, Düsseldorf, Germany; ^2^ Brain and Mind Center, University of Sydney, Sydney, NSW, Australia; ^3^ Department of Neurology, Medical University of Vienna, Vienna, Austria

**Keywords:** confocal scanning laser ophthalmoscopy, microglia, CX3CR1-GFP, experimental autoimmune encephalomyelitis, live-tracking

## Abstract

Confocal scanning laser ophthalmoscopy (cSLO) is a non-invasive technique for real-time imaging of the retina. We developed a step-by-step protocol for the semi-automatic evaluation of myeloid cells in cSLO images from CX3CR1^GFP^ mice, expressing green fluorescent protein (GFP) under control of the endogenous CX3C chemokine receptor 1 locus. We identified cSLO parameters allowing us to distinguish animals with experimental autoimmune encephalomyelitis (EAE) from sham-treated/naïve animals. Especially cell count (CC) and the total microglial area (SuA) turned out to be reliable parameters. Comparing the cSLO results with clinical parameters, we found significant correlations between the clinical EAE score and the SuA and of the inner retinal layer thickness, measured by optical coherence tomography, with the CC as well as the SuA. As a final step, we performed immunohistochemistry to confirm that the GFP-expressing cells visualized by the cSLO are Iba1 positive and validated the step-by-step protocol against manual counting. We present a semi-automatic step-by-step protocol with a balance between fast data evaluation and adequate accuracy, which is optimized by the option to manually adapt the contrast threshold. This protocol may be useful for numerous research questions on the role of microglial polarization in models of inflammatory and degenerating CNS diseases involving the retina.

## Introduction

The chemokine receptor and chemokine ligand axis mediates chemotaxis of immune cells. These mechanisms apply to immune but also non-immune cells throughout the whole body, but the exact expression pattern is cell type specific for the particular organ (1). In the central nervous system (CNS), the CX3C chemokine receptor 1 (CX3CR1) is mainly expressed on the developmentally yolk sac-derived microglial cells or, in pathological conditions, on infiltrating monocytes and macrophages, whereas chemokine ligands are predominantly expressed on neurons (2, 3). The higher the concentration of C-X3-C motif chemokine ligand 1 on cells, the more pronounced the chemotaxis of CX3CR1-bearing myeloid cells (4, 5). The importance of microglial polarization as an aspect in the pathogenesis of CNS diseases has increasingly moved in the focus of interest and has been studied specifically in ex-vivo models (6). Several preclinical studies have demonstrated that targeting the infiltrating monocyte-derived cells during the acute phase substantially reduces the severity of experimental autoimmune encephalomyelitis (EAE) (7–9), an animal model of multiple sclerosis (MS), while both detrimental (10) and beneficial (3) effects have been attributed to the resident microglia. In the remission phase, microglia may play an important role in regulating immune functions and restoring function by facilitating repair and remyelination, ultimately preventing chronic neurodegeneration. At the same time, chronic microglial activation may very well drive demyelination and neuroaxonal loss during progressive phases (11). Transcriptome analyses of single microglial cells from different regions of the CNS from demyelination models in mice have led to the identification of specific microglial clusters that appear to be associated with certain CNS diseases. Furthermore, it was possible to extract microglial clusters from human tissue of MS patients that display similarities with the clusters of the demyelinating mouse models (12). Overall, microglial activity, with its reactive oxygen species-mediated phagocytosis, but also with its cytokine- and chemokine-modulating function presents itself as a crucial aspect of the MS pathogenesis. It has been associated with blood-brain barrier permeability, dysregulated T-cell activation and modulating effects on B-cells (13–19). Using non-invasive, *in vivo* confocal scanning laser ophthalmoscopy (cSLO), microglial activation can also be assessed in the retina of CX3CR1^GFP^ mice in the context of EAE (20).

We therefore aimed to develop a protocol to facilitate the investigation of the dynamics of microglial activation in the myelin oligodendrocyte glycoprotein, fragment 35-55 (MOG_35-55_) EAE model, using cSLO as a non-invasive tool for live cell tracking in CX3CR1^GFP^ mice (21, 22). The specific aims of the study were to I.) further characterize the macroscopic dynamics of microglial activity in EAE; II.) develop a semi-automated method for *in vivo* tracking and III.) correlate the cSLO-based parameters with one another and against other readouts, such as the retinal thickness measured by optical coherence tomography (OCT) and histology.

We developed a detailed instruction to simplify and standardize the tracking of myeloid cell changes *in vivo*.

## Material and Methods

### Mouse Strain and Animal Housing

All experiments were performed in 6-week-old female and male heterozygous mice of strain B6.129P2(Cg)-Cx3cr1^tm1Litt^/J (The Jackson Laboratory, Stock No: 005582), hereafter referred to as CX3CR1^GFP^. Mice were bred in-house under standard conditions in temperature-controlled rooms under a 12/12 light/dark schedule, with lights on at 06:00 h until 18:00 h. In this strain, an enhanced green fluorescent protein (EGFP) sequence replaces the first 390 base pairs of the second CX3CR1 exon, which encodes the N-terminus of the seven G-coupled transmembrane receptors of the family (1, 4). Animal experiments were performed in compliance with the experimental guidelines approved by the regional authorities (State Agency for Nature, Environment and Consumer Protection; AZ 84-02.04.2014.A059, AZ 84-02.04.2016A137, AZ 81-02.04.2019.A063) and conform to the European directive 2010/63/EU on the protection of animals used for scientific purposes.

### Experimental Groups and EAE Immunization

The animals were divided in three experimental groups. In the first experimental group, we induced EAE by subcutaneous injection of 200 µg of myelin oligodendrocyte glycoprotein, fragment 35-55 (MOG_35-55_, Biotrend) emulsified in complete Freund’s adjuvant (CFA) in CX3CR1^GFP^mice as previously described (23). Two intraperitoneal injections of 200 ng of pertussis toxin (PTX, Sigma-Aldrich) followed on the same day and two days after immunization. For the second group, CX3CR1^GFP^ mice only received CFA and PTX serving as control group, hereafter called Sham-treated. The third group consisted of untreated CX3CR1^GFP^ mice (naïve group). To rate the EAE severity of the mice, we determined the clinical EAE score daily using the following criteria: (0) no disease; (0.5) mild tail paresis; (1) obvious tail paresis or plegia; (1.5) tail plegia and no righting reflex; (2) mild signs of hind limb paresis with clumsy gait; (2.5) obvious signs of hind limb paresis; (3) hind limb plegia, mouse drags one leg behind; (3.5) hind limb plegia, mouse drags both legs behind; (4) mild signs of quadriparesis; (4.5) quadriplegia; and (5) death or moribund (23, 24).

### Optical Coherence Tomography and Confocal Scanning Laser Ophthalmoscopy Acquisition

For *in vivo* imaging, we anesthetized the mice with isoflurane (Piramal critical care, Mumbai, India; 3% at 2L 02/min) using a vaporizer. We then wrapped the anesthetized animal in paper tissue to protect it from cooling and positioned it on the custom-made device. Eye drops with phenylephrine (2.5%)-tropicamide (0.5%) were administered to dilate the mouse pupils and were replaced after approximately one minute by Visc-Ophtal eye gel (Dr. Winzer, Berlin, Germany) to prevent corneal desiccation. Finally, we applied a custom-made contact lens (+4 diopters) to the eye before OCT and cSLO measurement for better image resolution as previously described (24–26).

OCT methodology is reported according to the updated APOSTEL recommendations (27, 28). Like in previous OCT studies (23, 24, 29, 30), we performed volume scans (25 x 25°) to determine retinal layer thicknesses using a Spectralis™ HRA+OCT device (Heidelberg Engineering, Germany) under ambient light conditions and excluded images with a quality below 20 decibels. We determined the thickness of the total retinal layer thickness (TRT) and of the inner retinal layer (IRL), which is composed of the retinal nerve fiber layer (RNFL), ganglion cell layer (GCL), and inner plexiform layer (IPL), using the automatic segmentation of the Heidelberg Eye Explorer™ software (version 1.9.10.0). Segmentation errors were corrected by a blinded examiner. Previous studies have demonstrated the usefulness of combining the three layers into the IRL (25, 30).

The Spectralis™ HRA+OCT device (Heidelberg Engineering, Germany) includes the option for cSLO (31–33), and measurement of the CX3CR-1^GFP^ mice was performed as described above following the OCT imaging. The HRA+OCT device was set to blue autofluorescence (BAF) mode (λ = 488 nm, barrier filter to capture fundus emissions above λ= 500 nm) (34), manually maximizing the image saturation while the reflected light image of the retina was averaged from 30 real-time reflected light images.

### Image Analysis and Semi-Automatic Step-By-Step Protocol

**Figure 1:**
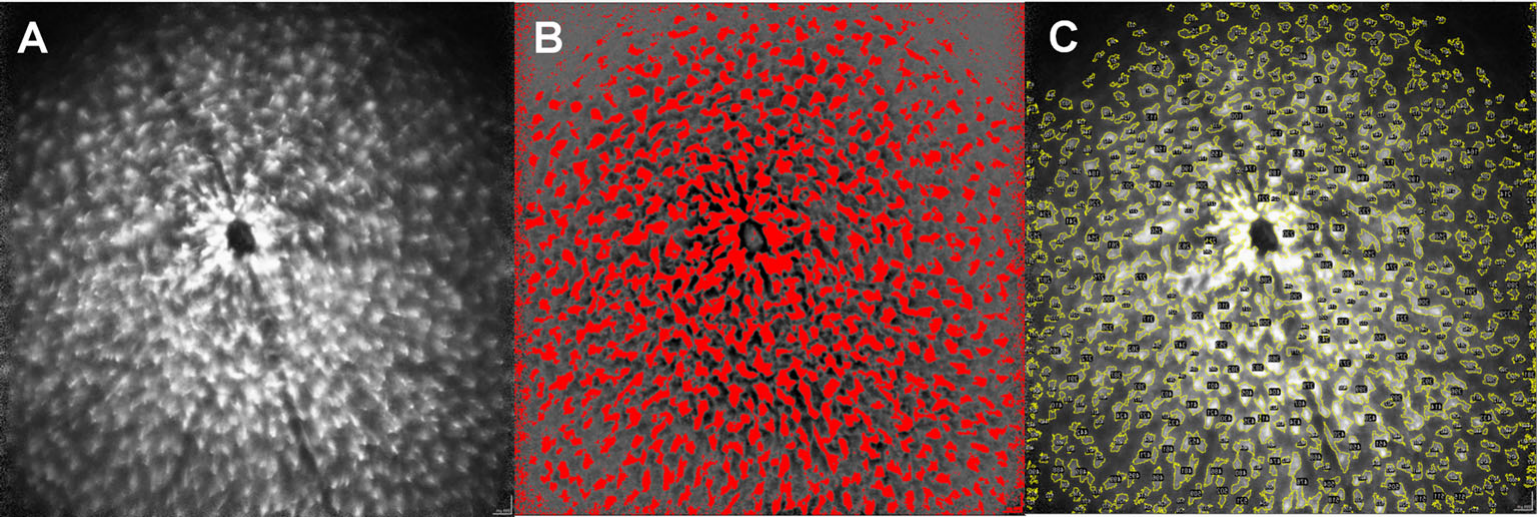
Step-by-step protocol for image analysis *via* ImageJ. Original image of a MOG-immunized mouse after 6 weeks **(A)**, semi-automatic generation of a mask for the corresponding image by enhancing the light-dark contrast and establishing a threshold of the coloration **(B)**. Color coded mapping of the mask to the original image to obtain quantitative values within the yellow colored areas **(C)**, numbered in the ImageJ application by structures here recognizable as black dots). The size of the cells, as well as different brightness parameters are considered by the step-by-step protocol to create the mask.

We developed a step-by-step instruction on how to semi-automatically analyze microglial dynamics *in vivo* using the open source Java image processing software ImageJ (version: 1.53g; provided in the public domain at https://imagej.nih.gov/ij/index.html by the National Institutes of Health, Bethesda, Maryland, USA). This two-part step-by-step instruction enables the transition of a mouse retina cSLO image (**Figure 1A**) into quantifiable parameters by ImageJ.

In the first step, we created a mask from the original image by increasing the contrast using ImageJ, which delineated the GFP expressing microglia cells from the darker background of the mouse retina. This was achieved by harmonizing the background using the ImageJ command “Substract Backround” setting the “rolling ball radius” to 500 pixels and then filtered the resulting image *via* a Fast Fourier Transform (ImageJ command: “FFT bandpass filter”), setting the limits for large structures at 30 pixels and for small structures at 3 pixels. We then manually adjusted the threshold (ImageJ command: “Threshold”) to delineate the microglial cells from the background (**Figure 1B**). As this is a step-wise process, the procedure requires an evaluator who is blinded for the experimental groups, especially to determine the separation threshold for microglia and the background. The threshold then serves as a verifiable criterion for the transformation of the qualitative picture into quantitative data.

In the second step, the mask obtained in step 1 is superimposed on the original image and the parameters area, maximum and minimum brightness, diameter of the area separated by the template and its Feret diameter can be obtained from the original image, using the mask (**Figure 1C**). A detailed step-by-step description with the standardized threshold values can be found in the **Supplementary Data**.

### Immunohistochemical Staining of Retinal Cross-Sections

In addition to the GFP signal from CX3CR1^GFP^ retinae, immunohistochemical Iba1 (ionized calcium binding adapter molecule 1) staining of retinal sections was used for histological detection of myeloid cells. After the mice were sacrificed, we confirmed that the GFP signal indeed indicates activated myeloid cells by additional Iba1 staining. A detailed protocol can be found in the **Supplementary Material**. In brief, the eyes were dissected from 6 representative animals per group 12 weeks after immunization and processed as previously described (35). After paraffin embedding, eyes were longitudinally sectioned at 5 µm and stained with Iba1 (1:500, Wako chemicals) and Cy5 anti-rabbit (1:500, Millipore) as secondary antibody. 40 retinal sections per mouse were analyzed.

### Statistical Analysis

Statistical analysis was performed using Prism 5.0 (Graphpad, San Diego, USA) and SPSS Statistics 20 (IBM, Endicott, USA). P-values were considered significant with p<0.025 resulting from a Bonferroni correction for multiple testing.

Linear Regression (Prism) was used to analyze the following correlations: I.) The correlation between manual and automated counting of cells by ImageJ, II.) the correlation between Iba1 histology and cSLO data at 12 weeks, III.) the correlation between the cSLO parameters total microglial area (SuA) and cell count (CC) and the clinical score, and IV.) the correlation between cSLO parameters [SuA, CC, Average cell brightness (MVI), Maximal cell brightness (MCB), Average cell area (AvA)] and the retinal thickness parameters (TRT, IRL) measured by OCT.

The area under the curve (AUC) was applied to assess the predictive power of the individual cSLO parameters (SuA, CC, MVI, MCB, AvA) with regard to the presence and severity of an active EAE. Each cSLO parameter was subsequently transformed into a Z-score, allowing the combination of individual parameters into a composite score from multiple parameters.

Differences of the cSLO and OCT parameters between means of the EAE, sham and naïve groups were analyzed using generalized estimating equations (GEE) with an exchangeable correlation matrix, considering the specification of the mouse eye (right or left) as a within-subject variable. The number of weeks after immunization was included as an additional within-subject variable when analyzing the association between OCT and cSLO parameters.

The differences between the microglial cell counts of the three treatment groups in Iba1 histology at the 12-week time point were investigated by one-factor ANOVA, followed by Bonferroni correction.

## Results

### The cSLO Parameters Separate EAE and Sham-Treated Animals

**Figure 2:**
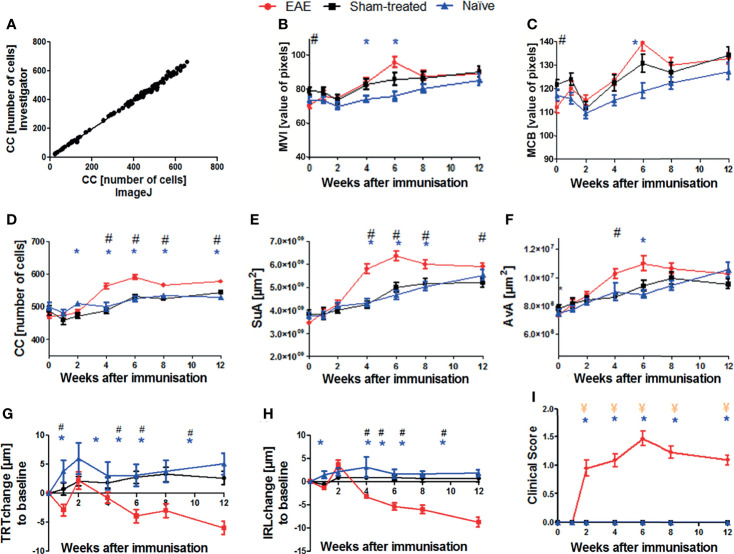
Validation of the step-by-step semi-automated ImageJ protocol and results of cSLO, OCT, and EAE clinical score parameters within the EAE, sham-treated, and naïve groups over 12 weeks. Correlation between cell count (CC) determined by retinal images from a blinded examiner and from the ImageJ step-by-step protocol **(A)**; n = 130, R^2^ = 0.997, p < 0.0001. Confocal-SLO parameters mean value intensity MVI **(B)**, maximum cell brightness MCB **(C)**, cell count CC **(D)**, sum of area SuA **(E)** and average cell area AvA **(F)**. OCT parameters change of total retinal thickness TRT **(G)** and inner retinal layer thickness IRL **(H)**. EAE clinical score **(I)**; **(B–I)** represent the pooled mean ± SEM; n = 75 (EAE n = 34, sham-treated n = 21, naïve n = 20); *p < 0.025 for EAE *vs* naïve, # p < 0.025 for EAE *vs* sham-treated and ¥ p < 0.025 for sham-treated *vs* naïve by GEE with Bonferroni correction.

To establish a quantitative image analysis using the ImageJ step-by-step protocol described above, we evaluated 130 cSLO-CX3CR1^GFP^ retinal images manually determining the cell number for each image by a blinded investigator (gold standard) and by a second investigator using the new step-by-step protocol. A Pearson correlation of the cell counts obtained by both approaches revealed an excellent correlation coefficient of R^2^ 0.997 (p<0.001, **Figure 2A**), with results ranging from 22 to 660 cells across all images.

We then analyzed the cSLO images of the EAE (n = 34), sham (n = 21), and naïve (n = 20) CX3CR-1^GFP^ mice to capture the cSLO parameters at weeks 0, 1, 2, 4, 6, 8 and 12 after immunization.

The most profound changes for these measurements were observed in weeks 4, 6, and 8, the peak of each cSLO parameter was reached in week 6 (**Figures 2B–F**). While the parameter values did not differ significantly between the three treatment groups at the beginning and at the end of the observational period, significant differences were observed in weeks 4 to 8, especially for the CC and SuA. EAE mice showed the highest total values, while sham-treated and naïve animals presented similar results with a significant difference to EAE mice (p<0.025) (**Figures 2D, E**). The brightness parameters MVI and MCB did not reveal a significant difference between the experimental groups with the exception of the baseline measurements of the sham-treated compared to the MOG_35-55_ immunized mice (**Figures 2B, C**). The AvA reflected the results of the CC and SuA, however, without reaching significant levels for naïve/sham-treated mice compared to EAE mice for the different timepoints (**Figure 2F**).

While the IRL thickness and TRT of sham-treated and naïve mice remained almost constant, the retinal thickness of EAE mice slightly decreased initially, followed by retinal swelling at week 2 and then continuously progressing degeneration over the course of the experiment. The thickness change of the EAE animals differed significantly compared to the baseline measurement and differed significantly (p<0.025) compared to the two other groups from week 4 onwards. (**Figures 2G, H**).

Starting at week 2, EAE mice presented progressive EAE disability scores, reaching a peak at week 6, and a slow recovery with a rather chronic progression until the end of the experiment. Sham-treated and naïve mice did not develop a clinical score throughout the experiment, while the MOG-immunized group presented significantly higher clinical EAE scores (p<0.025; **Figure 2I**).

### The Cell Count and the Sum of Area Reflect Microglial Activation

**Figure 3:**
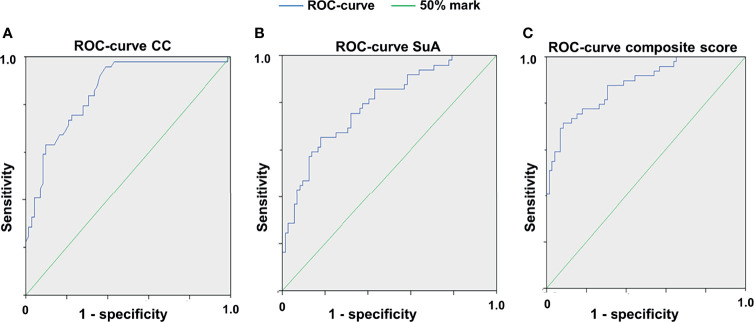
Receiver operating characteristic curves for the different cSLO parameters. ROC for CC **(A)**, for SuA **(B)** and for the composite score combined from the two parameters **(C)**, 6 weeks after immunization; n = 75, p < 0,025, with Bonferroni correction. The composite score **(C)** achieves a higher diagnostic discrimination, as shown by the larger area under the curve (AUC) indicating higher sensitivity and specificity.

Since microglial activation has been associated with retinal degeneration in inflammatory CNS disorders (11, 36) we addressed the question to what extent the cSLO parameters CC and SuA can differentiate MOG- from sham-treated or naïve mice. For this purpose, we calculated receiver operating characteristics (ROCs) of the test variables CC and SuA for the variable MOG immunization (**Figures 3A, B**). Based on the AUC for each week the CC (AUC week (W) 4: 77.85%, W6: 85.95%, W8: 73.05%, W12: 75.85% - for all values p< 0.025) proved to be slightly better suited to separate the groups than the SuA (AUC W4: 77.8%, W6: 79.2%, W8: 68.45%, W12: 64.5% - for all values p<0.025). However, the combination of both parameters to a composite score resulted in an increase of the diagnostic discriminatory power compared to each of the criteria alone (AUC W6: 88.2% > W4: 81.4% > W8: 77.0% > W12: 75.0% - for all values p<0.025), explaining 88.2% (W6) of all cases in the best case and 75% (W12) in the worst case (**Figure 3C**). In summary, depending on the requirements for sensitivity or specificity, a cut-off of 0.6547 for week 6 (sensitivity 77.6%, specificity 81.9%, see also **Table S2**) can be determined using the composite score combining the CC and SuA, which allows the separation of EAE immunization against sham-treated/naïve mice based on a retinal fundus image with fluorescence-labeled cells. For week 8 and 12 we determined a cut-off value of 0.439 (week 8, sensitivity 71.6%, specificity 69.5%) and 0.5704 (week 12, sensitivity 72.3%, specificity 67.1%) respectively in order to achieve a balanced ratio of sensitivity and specificity. By modifying the cut-off values, the ratio of sensitivity and specificity can be adapted, depending on the requirements of the individual researchers (see **Tables S2, S4, S6** for cut-off values with corresponding sensitivities and specificities in the **Supplementary Material**).

### Retinal Degeneration Correlates With cSLO Readouts

**Figure 4:**
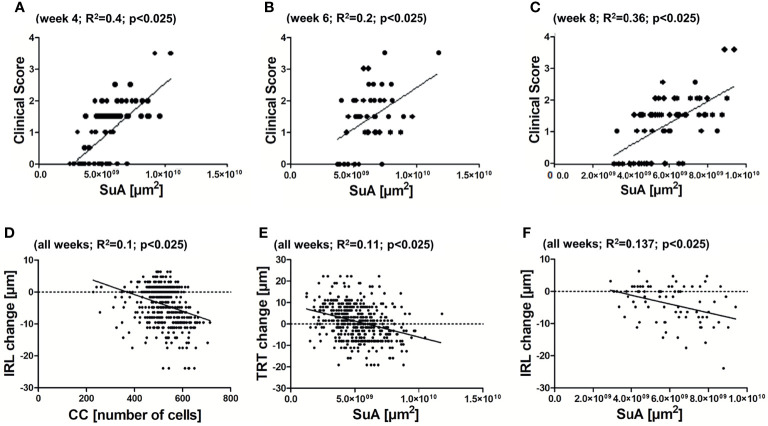
Correlations between cSLO and OCT parameters. Positive correlation between SuA and clinical scoring of the EAE group at 4 weeks **(A)** - R^2^ = 0.4; p < 0.025), 6 weeks **(B)** - R^2 ^= 0.2; p < 0.025), and 8 weeks **(C)** - R^2 ^= 0.36; p < 0.025) after immunization. Negative correlation between CC and IRL **(D)** - R^2 ^= 0.1; p < 0.025) and SuA and TRT **(E)** - R^2 ^= 0.11; p < 0.025) of all weeks. Non-significant association between SuA and IRL **(F)** - R^2 ^= 0.137; p > 0.025), with Bonferroni correction.

We then investigated the correlations between the cSLO parameters and the clinical score as well as the OCT readouts. For both correlations, only the cSLO parameters SuA and CC were taken into account, as only these two revealed significant differences between the treatment groups. The correlation analysis of cSLO with the clinical score for the period of maximum microglial activation from week 4 to 8, only revealed a positive correlation with the clinical EAE score for the parameter SuA (R^2^ between 0.2 and 0.4, p<0.025, **Figures 4A–C**). Only immunized EAE mice were included, as the other groups presented no clinical score (**Figure 2I**). Investigating the retinal layer thickness measured by OCT, SuA and CC revealed significant negative correlations over all weeks: When the CC increased, the IRL thickness decreased (R^2^ = 0.1, p<0.025; **Figure 4D**); when the SuA increased, the TRT also decreased (R^2^ = 0.11, p<0.025; **Figure 4E**). In summary, the SuA correlated positively with the clinical scoring of the mice and negatively with the TRT, while the CC correlated negatively with the inner retinal layer thickness (**Figure 4**).

### The Semi-Automated Step-By-Step Protocol Can Be Validated by Histological Analysis

**Figure 5:**
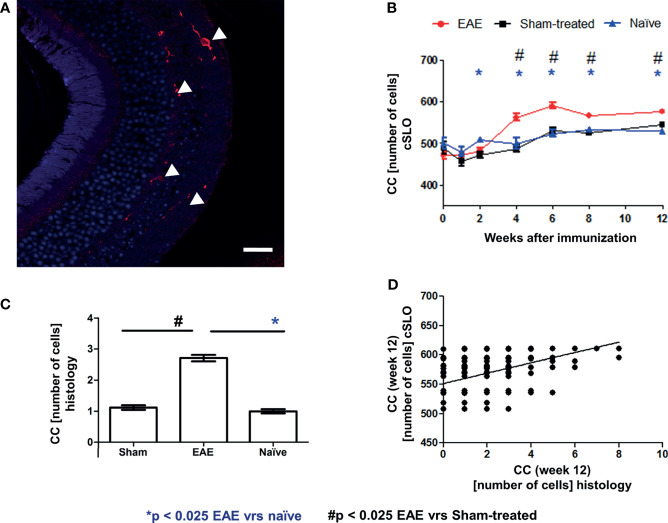
Correlation of cSLO images and Iba1 histology validated cell count at 12 weeks after immunization. Iba1 stained retinal paraffin section of an EAE mouse after 12 weeks **(A)** - (Confocal microscope, x63 enlargement: Iba1 stained cells marked by indicator arrows, scale bar = 25 µm). In both cSLO **(B)** and Iba1 staining **(C)**, the EAE group shows the highest cell number, while sham-treated and naïve groups present nearly equal microglial cell numbers and differ significantly from the EAE group in histology. The bar graphs are presented as mean ± SEM with *p < 0.025 for EAE vrs naïve and # p < 0.025 for EAE vrs sham-treated by one-factor ANOVA, followed by Bonferroni correction. **(C)**. The microglial cell count from the histological staining (n = 720) positively correlates with the cSLO images [**(D)** - R^2^ = 0.177, p < 0.001)]; with *p < 0.025 for EAE *vs* naïve and # p < 0.025 for EAE *vs* sham-treated by GEE with Bonferroni correction.

As a final confirmatory step, we validated the microglial cSLO readouts by a histological assessment. For this purpose, retinae of 18 representative mice (6 EAE, 6 sham-treated, 6 naïve mice) were immunohistochemically stained with Iba1 12 weeks after immunization and evaluated by a blinded observer (**Figure 5A**). Differences in microglial cell number at 12 weeks were analyzed by ANOVA. The cSLO imaging revealed significantly higher cell numbers in the EAE group compared to the other two groups (**Figure 5B**). In line with these findings, the histological assessment revealed higher microglial cell numbers in the EAE animals compared to the sham-treated and naïve mice, which did not differ between each other, confirming the results of the cSLO measurement (**Figure 5C**). A positive correlation between the cell count in the cSLO image and the histological analysis was demonstrated at 12 weeks after immunization (R^2^ = 0.177 p<0.025, **Figure 5D**).

## Discussion

The retina represents an easily accessible compartment of the CNS for *in vivo* imaging by OCT as well as fundus imaging and live-cell tracking by cSLO (26, 37). Longitudinally tracking the microglial cSLO outcomes revealed a steadily increasing CC and SuA of all experimental groups over the period of 12 weeks, although the sham-treated and naïve mice were not MOG_35-55_ immunized. Of note, the CC and SuA were significantly lower in naïve and sham mice compared to EAE animals in the period of maximum microglial activation (4-8 weeks post immunization). It seems reasonable to assume that the increase in microglial cell numbers and cell area in non-immunized animals can be explained by the maturation of the animals (38). This should be taken into account when planning experiments focusing on retinal microglia dynamics in mice of this age. In addition, the genetic background of the animal strain has to be considered when performing experiments on brain myeloid cells, as it was demonstrated that genetic diversity significantly alters features and dynamics of microglia, already in baseline neuroimmune functions (39, 40). Therefore, these results obtained in mice with a C57BL6J background may not be transferable and will have to be confirmed in other mouse strains.

While the cSLO measurements presented a constant increase in almost all parameters, the time courses of the retinal thickness change measured by OCT showed an initial thickness loss one week after immunization in EAE mice suggesting retinal damage by early inflammatory processes. The thickness increase at week 2 can be explained by edema linked to early microglial activation and astrogliosis. The following decrease of the total retinal thickness and IRL reflects the chronic neurodegeneration after axonal demyelination and the acute inflammatory insult (30, 41).

Technically, the challenge of keeping the optic nerve head focused and light saturation exactly identical during the measurement and across the different time points of the mice arose during the optimization process of the study (36). The image evaluation using cSLO is strongly influenced by parameters such as image illumination and the laser angle on the retina of the examined animal, even leading to significant differences between the baseline brightness values of the sham-treated and EAE mice. In laboratory practice, it proved to be extremely difficult to keep these parameters exactly identical over repeated measurement sessions spanning several weeks. Therefore, we decided to include an adaptable threshold for evaluation of the images. After the sub-step of contrasting the microglia from the background, a threshold was manually set to separate the microglial cell area from the background by means of the mask (see **Figure 1B**). This approach of semi-automated segmentation allowed the evaluator to compensate for variations in illumination or focus. At the same time, it is a potential source of bias. This is why it is of the utmost importance, that the investigator is blinded for the experimental groups. The step-by-step protocol was validated by histological retinal sections stained with Iba1, which were manually counted and showed a significant correlation with the results obtained by the cSLO.

Other approaches opted for a fully automated segmentation using constant thresholds at this point of data acquisition (36, 42, 43). To delineate the microglia by a constant threshold, a fixed algorithm is applied based on parameters such as the cell morphology to decide whether a microglial cell is “activated” or not (36, 43). However, inaccurate and inconsistent saturation and focus settings can lead to artefacts in the GFP detection when evaluating the images by a fully automatic algorithm without the possibility of correction. The semi-automatic step-by-step protocol presented here retains the possibility of correction by the blinded observer, but it is more time-consuming than a fully automatic algorithm. In order to make it more time-efficient, future research should focus on developing a software tool for automating the microglia masking process.

The main challenges for the design of an automatic step-by-step protocol to evaluate the cSLO-CX3CR-1^GFP^ images also arose from the morphological changes of microglial cells during activation. Upon activation, the cells shift from small cell somata with long, ramified branches to an amoeba-like structure with short cell processes (40, 42–44). This morphology can easily be identified in histological sections, but may be challenging to detect in cSLO images depending on the image focus and overlay of other microglial cells during massive infiltration in the context of EAE (42). However, compared to histology, the cSLO-method allows to investigate the retinal microglial activation non-invasively and longitudinally, reducing the number of animals required, avoiding the inter-subject variability and opening up opportunities to react promptly to the dynamics of an experiment.

Other researchers used cSLO to investigate the morphological changes of microglial cells in non-inflammatory models (45). Different and possibly fully automated algorithms may be applicable to such models where microglial activation and myeloid cell infiltration is less intense than it is in MOG EAE where the huge number of microglia cells per image with overlapping somata make the differentiation of individual shapes extremely difficult. Therefore, in inflammatory models, semi-automatic tracking by cSLO still requires histological supplementation for morphological conclusions.

Optomotor response (OMR) is a method for investigating clinical changes of the visual system which uses the optokinetic nystagmus of the animals (24, 42). This could provide a closer link between clinical and microglial readouts in future studies than is achieved with the clinical score and its focus on locomotor change.

In summary, we identified cSLO parameters, which can help to discriminate EAE animals from sham-treated/naïve mice based on retinal fundus images with CX3CR1-GFP fluorescence-labeled cells. CC and SuA were increased in EAE and revealed correlations with the retinal thickness changes measured by OCT and with the clinical score. Our semi-automated step-by-step protocol can be helpful for numerous research questions and may contribute to further decipher the role of microglial polarization in models of inflammatory and degenerating CNS diseases involving the retina.

## Data Availability Statement

The original contributions presented in the study are included in the article/**Supplementary Material**. Further inquiries can be directed to the corresponding author.

## Ethics Statement

The animal study was reviewed and approved by State Agency for Nature, Environment and Consumer Protection; AZ 84-02.04.2014.A059, AZ 84-02.04.2016A137, AZ 81-02.04.2019.A063.

## Author Contributions

MF, CH, AI, and MD performed the experiments and analyzed the data. MF, MD, and PA wrote the manuscript. H-PH, SM, and MS were involved in revising the manuscript critically for important intellectual content and made substantial contributions to interpretation of data. PA and MD conceived the study and supervised experiments. All authors read and approved the final manuscript.

## Funding

This work was supported by grants from the charitable Ilselore-Lückow Stiftung and the charitable Dr.-Robert-Pfleger Stiftung to PA.

## Conflict of Interest

The following financial disclosures are unrelated to the work: H-PH has received fees for serving on steering and data monitoring committees from Bayer Healthcare, Biogen, Celgene BMS, CSL Behring, GeNeuro, MedImmune, Merck, Novartis, Octapharma, Roche, Sanofi Genzyme, TG Therapeutic sand Viela Bio; fees for serving on advisory boards from Biogen, Sanofi Genzyme, Merck, Novartis, Octapharma, and Roche; and lecture fees from Biogen, Celgene BMS, Merck, Novartis, Roche, Sanofi Genzyme. SM received honoraria for lecturing and travel expenses for attending meetings from Almirall, Amicus Therapeutics Germany, Bayer Health Care, Biogen, Celgene, Diamed, Genzyme, MedDay Pharmaceuticals, Merck Serono, Novartis, Novo Nordisk, ONO Pharma, Roche, Sanofi-Aventis, Chugai Pharma, QuintilesIMS, and Teva. His research is funded by the German Ministry for Education and Research (BMBF), Deutsche Forschungsgemeinschaft (DFG), Else Kröner Fresenius Foundation, German Academic Exchange Service, Hertie Foundation, Interdisciplinary Center for Clinical Studies (IZKF) Muenster, German Foundation Neurology, and by Almirall, Amicus Therapeutics Germany, Biogen, Diamed, Fresenius Medical Care, Genzyme, Merck Serono, Novartis, ONO Pharma, Roche, and Teva. MD received speaker honoraria from Merck. PA received compensation for serving on Scientific Advisory Boards for Ipsen, Novartis, Biogen; he received speaker honoraria and travel support from Novartis, Teva, Biogen, Merz Pharmaceuticals, Ipsen, Allergan, Bayer Healthcare, Esai, UCB and Glaxo Smith Kline; he received research support from Novartis, Biogen, Teva, Merz Pharmaceuticals, Ipsen, and Roche.

The remaining authors declare that the research was conducted in the absence of any commercial or financial relationships that could be construed as a potential conflict of interest.

## Publisher’s Note

All claims expressed in this article are solely those of the authors and do not necessarily represent those of their affiliated organizations, or those of the publisher, the editors and the reviewers. Any product that may be evaluated in this article, or claim that may be made by its manufacturer, is not guaranteed or endorsed by the publisher.

## Abbreviations

AUC, Area under the curve; AvA, Average cell area; BAF, Blue auto fluorescence; CC, Cell count; CFA, complete Freund’s adjuvants; CNS, Central nervous system; cSLO, confocal scanning laser ophthalmoscopy; CX3CR1, CX3C chemokine receptor 1; EAE, Experimental autoimmune encephalomyelitis; (E)GFP, (Enhanced) green fluorescent protein; IBA1, Ionized calcium-binding adaptor molecule 1; IRL, Inner retinal layers; MCB, Maximal cell brightness; MOG, Myelin oligodendrocyte glycoprotein; MS, Multiple sclerosis; MVI, Average cell brightness; OMR, Optomotor response; PTX, Pertussis toxin; SuA, Total microglial area; TRT, Total retinal thickness.
