# Supplementary material for: Semi-Automated Live Tracking of Microglial Activation in CX3CR1GFP Mice During Experimental Autoimmune Encephalomyelitis by Confocal Scanning Laser Ophthalmoscopy
Source: Front Immunol. 2021 Oct 21;12:761776. doi: 10.3389/fimmu.2021.761776 (PMC8567040; doi:10.3389/fimmu.2021.761776)
Supplement: Supplementary file 1 [file DataSheet_1.docx]

***Supplementary material***

**1 Supplementary Data**

**1.1 Protocol of immunohistochemical Iba1 staining of paraffin sections**

Xylol (10 min), Xylol (5 min), 96% - 80 % - 70 % - 60 % Ethanol for 4 minutes each, PBS (phosphate-buffered saline, 5 min), 10 nM Citrate buffer (2,94g Na-citrat-dihydrat/1l+0,5 ml Tween 20 ph 6) 10 min in microwave. PBS 5 min, 1% Triton X-100 5 min, PBS 5 min, border paraffin sections with Dako Pen, 2% BSA (Bovine serum albumin) in PBS 2 h, primary antibody Iba1 (rabbit) 1:500 in 2% BSA 24 h. Washing three times in PBS for 5 min each time, second antibody Cy3 (goat anti rabbit) 1:500 in 2% 1h, BSA 24 h, washing three times in PBS for 5 min each time, DAPI 1:100 in PBS 5 min, washing two times in PBS for 5 min each time, cover in immomount and store at 4 degrees.

**1.2 ImageJ supported semi-automatic step-by-step protocol**

1. Open ImageJ

2. Select Image/Type/8bit

3. Measure scale with ruler/Analyse/Set Scale (refer µm + click "global"!)

4. Select Process/Substract Background (500 pixels)

5. Select Process/FFT/BandpassFilter (large structures: 30 pixels/small structures: 3 pixels)

6. Select Image/Adjust/Threshold/Apply (Threshold flexible - always note!)

7. Select Process/Binary/Convert to mask

8. Select Analyze/Set Measurements (Items: Area, Min/max, Mean, Perimeter, Standard Deviation, Centroid, Feret)

9. Frame the area to be analysed excluding the image scale

10. Select Analyze Particles (150 Pixel Junits)

11. Close edited mask and open the original (then select: show all)

12. Measure the microglia area of the original image marked by the mask

**1.3 Four-field tables and cut-off values**

**Table S1: Cut-off values for week 6 with corresponding sensitivity and specificity.**

| Cut-off of 0,6547  (Week 6,  sensitivity 77.6 %, specificity 81.9 %) | EAE immunization | Sham-treated/naïve mice |  |
| --- | --- | --- | --- |
| Positive score | 35.2 %  (true positives) | 8.0 %  (false positives) | 43.2 % |
| Negative score | 10.1 %  (false negatives) | 46.7 %  (true negatives) | 56.8 % |
|  | 45.3 % | 54.7 % | 100.00 % |

**Table S2: Cut-off values and coordinates of the curve week 6.**

| Variable(s) for test result: composite score | | |
| --- | --- | --- |
| Cut-off values | sensitivity | 1 - specificity |
| ,0000 | ,959 | ,597 |
| ,0022 | ,959 | ,583 |
| ,0389 | ,959 | ,569 |
| ,1077 | ,939 | ,569 |
| ,1558 | ,939 | ,556 |
| ,1743 | ,939 | ,542 |
| ,1856 | ,918 | ,542 |
| ,1959 | ,918 | ,528 |
| ,2150 | ,918 | ,514 |
| ,2407 | ,918 | ,500 |
| ,2501 | ,918 | ,486 |
| ,2533 | ,918 | ,472 |
| ,2608 | ,918 | ,458 |
| ,2737 | ,918 | ,444 |
| ,2844 | ,898 | ,444 |
| ,2887 | ,898 | ,431 |
| ,2979 | ,898 | ,417 |
| ,3146 | ,898 | ,403 |
| ,3346 | ,898 | ,389 |
| ,3551 | ,878 | ,389 |
| ,3763 | ,878 | ,375 |
| ,3977 | ,878 | ,361 |
| ,4135 | ,878 | ,347 |
| ,4466 | ,878 | ,333 |
| ,4814 | ,878 | ,319 |
| ,4954 | ,878 | ,306 |
| ,5156 | ,857 | ,306 |
| ,5310 | ,837 | ,306 |
| ,5463 | ,816 | ,306 |
| ,5601 | ,816 | ,292 |
| ,5634 | ,796 | ,292 |
| ,5721 | ,796 | ,278 |
| ,5782 | ,796 | ,264 |
| ,5803 | ,776 | ,264 |
| ,5838 | ,776 | ,250 |
| ,5869 | ,776 | ,236 |
| ,6051 | ,776 | ,222 |
| ,6310 | ,776 | ,208 |
| ,6406 | ,776 | ,194 |
| ,6547 | ,776 | ,181 |
| ,6716 | ,755 | ,181 |
| ,6783 | ,755 | ,167 |
| ,6875 | ,755 | ,153 |
| ,6994 | ,735 | ,153 |
| ,7195 | ,735 | ,139 |
| ,7457 | ,735 | ,125 |
| ,7729 | ,714 | ,125 |
| ,7972 | ,714 | ,111 |
| ,8070 | ,714 | ,097 |
| ,8326 | ,714 | ,083 |
| ,8645 | ,694 | ,083 |
| ,8973 | ,694 | ,069 |
| ,9333 | ,673 | ,069 |
| ,9620 | ,653 | ,069 |
| ,9819 | ,633 | ,069 |
| ,9839 | ,612 | ,069 |
| ,9863 | ,592 | ,069 |
| ,9952 | ,592 | ,056 |
| 1,0115 | ,592 | ,042 |
| 1,0295 | ,571 | ,042 |
| 1,0456 | ,551 | ,042 |
| 1,0537 | ,551 | ,028 |
| 1,0637 | ,531 | ,028 |
| 1,0736 | ,510 | ,028 |
| 1,0748 | ,510 | ,014 |
| 1,0839 | ,490 | ,014 |
| 1,1099 | ,469 | ,014 |
| 1,1495 | ,449 | ,014 |
| 1,1728 | ,429 | ,014 |
| 1,1872 | ,408 | ,014 |
| 1,2037 | ,408 | ,000 |
| 1,2247 | ,388 | ,000 |
| 1,2534 | ,367 | ,000 |
| 1,2711 | ,347 | ,000 |
| 1,3562 | ,327 | ,000 |
| 1,4535 | ,306 | ,000 |
| 1,4864 | ,286 | ,000 |
| 1,5019 | ,265 | ,000 |
| 1,5259 | ,245 | ,000 |
| 1,5581 | ,224 | ,000 |
| 1,5694 | ,204 | ,000 |
| 1,5846 | ,184 | ,000 |
| 1,6061 | ,163 | ,000 |
| 1,6347 | ,143 | ,000 |
| 1,6685 | ,122 | ,000 |
| 1,7105 | ,102 | ,000 |
| 1,7598 | ,082 | ,000 |
| 1,7978 | ,061 | ,000 |
| 1,8475 | ,041 | ,000 |
| 1,9201 | ,020 | ,000 |
| 2,9603 | ,000 | ,000 |

**Table S3: Cut-off values for week 8 with corresponding sensitivity and specificity.**

| Cut-off of 0,4390  (Week 8,  sensitivity 71.6 %, specificity 69.5 %) | EAE immunization | Sham-treated/naïve mice |  |
| --- | --- | --- | --- |
| Positive score | 32.4 %  (true positives) | 16.7 %  (false positives) | 49.1 % |
| Negative score | 12.9 %  (false negatives) | 38.0 %  (true negatives) | 50.9 % |
|  | 45.3 % | 54.7 % | 100.00 % |

**Table S4: Cut-off values and coordinates of the curve week 8.**

| Variable(s) for test result: composite score | | |
| --- | --- | --- |
| Cut-off values | sensitivity | 1 - specificity |
| ,0357 | ,896 | ,695 |
| ,0697 | ,896 | ,683 |
| ,0829 | ,896 | ,671 |
| ,1004 | ,896 | ,659 |
| ,1069 | ,881 | ,659 |
| ,1160 | ,881 | ,646 |
| ,1290 | ,881 | ,634 |
| ,1369 | ,866 | ,634 |
| ,1524 | ,866 | ,622 |
| ,1677 | ,866 | ,610 |
| ,1781 | ,866 | ,598 |
| ,1894 | ,851 | ,598 |
| ,1991 | ,851 | ,585 |
| ,2052 | ,851 | ,573 |
| ,2107 | ,851 | ,561 |
| ,2186 | ,851 | ,549 |
| ,2283 | ,851 | ,537 |
| ,2418 | ,851 | ,524 |
| ,2621 | ,851 | ,512 |
| ,2763 | ,851 | ,500 |
| ,2798 | ,851 | ,488 |
| ,2837 | ,851 | ,476 |
| ,2863 | ,851 | ,463 |
| ,2931 | ,851 | ,451 |
| ,3027 | ,836 | ,451 |
| ,3092 | ,836 | ,439 |
| ,3171 | ,836 | ,427 |
| ,3231 | ,821 | ,427 |
| ,3247 | ,821 | ,415 |
| ,3317 | ,821 | ,402 |
| ,3410 | ,821 | ,390 |
| ,3565 | ,806 | ,390 |
| ,3685 | ,806 | ,378 |
| ,3712 | ,791 | ,378 |
| ,3880 | ,791 | ,366 |
| ,4024 | ,791 | ,354 |
| ,4037 | ,776 | ,354 |
| ,4082 | ,761 | ,354 |
| ,4180 | ,746 | ,354 |
| ,4243 | ,746 | ,341 |
| ,4278 | ,731 | ,341 |
| ,4324 | ,731 | ,329 |
| ,4336 | ,716 | ,329 |
| ,4360 | ,716 | ,317 |
| ,4390 | ,716 | ,305 |
| ,4402 | ,701 | ,305 |
| ,4476 | ,687 | ,305 |
| ,4625 | ,687 | ,293 |
| ,4747 | ,672 | ,293 |
| ,4821 | ,657 | ,293 |
| ,4938 | ,642 | ,293 |
| ,5031 | ,627 | ,293 |
| ,5065 | ,627 | ,280 |
| ,5195 | ,612 | ,280 |
| ,5364 | ,612 | ,268 |
| ,5441 | ,612 | ,256 |
| ,5563 | ,597 | ,256 |
| ,5770 | ,597 | ,244 |
| ,5918 | ,597 | ,232 |
| ,6017 | ,597 | ,220 |
| ,6077 | ,597 | ,207 |
| ,6201 | ,597 | ,195 |
| ,6364 | ,582 | ,195 |
| ,6481 | ,567 | ,195 |
| ,6679 | ,567 | ,183 |
| ,6810 | ,567 | ,171 |
| ,6854 | ,552 | ,171 |
| ,6908 | ,537 | ,171 |
| ,6957 | ,537 | ,159 |
| ,7007 | ,537 | ,146 |
| ,7030 | ,522 | ,146 |
| ,7055 | ,507 | ,146 |
| ,7115 | ,507 | ,134 |
| ,7274 | ,493 | ,134 |
| ,7510 | ,478 | ,134 |
| ,7662 | ,463 | ,134 |
| ,7704 | ,463 | ,122 |
| ,7788 | ,448 | ,122 |
| ,7906 | ,448 | ,110 |
| ,7950 | ,433 | ,110 |
| ,7958 | ,433 | ,098 |
| ,8066 | ,433 | ,085 |
| ,8298 | ,433 | ,073 |
| ,8434 | ,418 | ,073 |
| ,8490 | ,418 | ,061 |
| ,8564 | ,403 | ,061 |
| ,8818 | ,388 | ,061 |
| ,9126 | ,373 | ,061 |
| ,9314 | ,373 | ,049 |
| ,9511 | ,358 | ,049 |
| ,9651 | ,358 | ,037 |
| ,9804 | ,358 | ,024 |
| 1,0058 | ,343 | ,024 |
| 1,0658 | ,328 | ,024 |
| 1,1291 | ,313 | ,024 |
| 1,1592 | ,299 | ,024 |
| 1,1744 | ,284 | ,024 |
| 1,1950 | ,269 | ,024 |
| 1,2451 | ,254 | ,024 |
| 1,2836 | ,254 | ,012 |
| 1,2905 | ,239 | ,012 |
| 1,2992 | ,239 | ,000 |
| 1,3310 | ,224 | ,000 |
| 1,3582 | ,209 | ,000 |
| 1,3735 | ,194 | ,000 |
| 1,3906 | ,179 | ,000 |
| 1,4079 | ,164 | ,000 |
| 1,4228 | ,149 | ,000 |
| 1,4292 | ,134 | ,000 |
| 1,4348 | ,119 | ,000 |
| 1,4947 | ,104 | ,000 |
| 1,5676 | ,090 | ,000 |
| 1,5940 | ,075 | ,000 |
| 1,6199 | ,060 | ,000 |
| 1,6630 | ,045 | ,000 |
| 1,7765 | ,030 | ,000 |
| 1,9484 | ,015 | ,000 |
| 3,0360 | ,000 | ,000 |

**Table S5: Cut-off values for week 12 with corresponding sensitivity and specificity.**

| Cut-off of 0,5704  (Week 12,  sensitivity 72.3 %, specificity 67.1 %) | EAE immunization | Sham-treated/naïve mice |  |
| --- | --- | --- | --- |
| Positive score | 32.8 %  (true positives) | 18.0 %  (false positives) | 50.8 % |
| Negative score | 12.5 %  (false negatives) | 36.7 %  (true negatives) | 49.2 % |
|  | 45.30 % | 54.70 % | 100.00 % |

**Table S6: Cut-off values and coordinates of the curve week 12.**

| Variable(s) for test result: composite score | | |
| --- | --- | --- |
| Cut-off values | sensitivity | 1 - specificity |
| ,0207 | ,923 | ,768 |
| ,0455 | ,923 | ,756 |
| ,0466 | ,923 | ,744 |
| ,0593 | ,923 | ,732 |
| ,0753 | ,923 | ,720 |
| ,0878 | ,923 | ,707 |
| ,1020 | ,923 | ,695 |
| ,1085 | ,923 | ,683 |
| ,1197 | ,908 | ,683 |
| ,1415 | ,908 | ,671 |
| ,1719 | ,908 | ,659 |
| ,1973 | ,892 | ,659 |
| ,2138 | ,892 | ,646 |
| ,2375 | ,892 | ,634 |
| ,2517 | ,892 | ,622 |
| ,2537 | ,877 | ,622 |
| ,2631 | ,877 | ,610 |
| ,2704 | ,877 | ,598 |
| ,2736 | ,862 | ,598 |
| ,2784 | ,846 | ,598 |
| ,2852 | ,846 | ,585 |
| ,2907 | ,846 | ,573 |
| ,3013 | ,846 | ,561 |
| ,3230 | ,846 | ,549 |
| ,3373 | ,846 | ,537 |
| ,3406 | ,846 | ,524 |
| ,3454 | ,846 | ,512 |
| ,3647 | ,846 | ,500 |
| ,3898 | ,846 | ,488 |
| ,4077 | ,846 | ,476 |
| ,4176 | ,846 | ,463 |
| ,4224 | ,831 | ,463 |
| ,4252 | ,831 | ,451 |
| ,4255 | ,815 | ,451 |
| ,4455 | ,800 | ,451 |
| ,4733 | ,800 | ,439 |
| ,4847 | ,800 | ,427 |
| ,4890 | ,785 | ,427 |
| ,4952 | ,785 | ,415 |
| ,5076 | ,769 | ,415 |
| ,5155 | ,754 | ,415 |
| ,5164 | ,738 | ,415 |
| ,5170 | ,738 | ,402 |
| ,5199 | ,723 | ,402 |
| ,5227 | ,723 | ,390 |
| ,5233 | ,723 | ,378 |
| ,5281 | ,723 | ,366 |
| ,5351 | ,723 | ,354 |
| ,5535 | ,723 | ,341 |
| ,5704 | ,723 | ,329 |
| ,5748 | ,708 | ,329 |
| ,5877 | ,692 | ,329 |
| ,6043 | ,677 | ,329 |
| ,6135 | ,677 | ,317 |
| ,6183 | ,662 | ,317 |
| ,6250 | ,646 | ,317 |
| ,6367 | ,631 | ,317 |
| ,6455 | ,631 | ,305 |
| ,6512 | ,631 | ,293 |
| ,6582 | ,631 | ,280 |
| ,6624 | ,615 | ,280 |
| ,6672 | ,600 | ,280 |
| ,6728 | ,585 | ,280 |
| ,6814 | ,585 | ,268 |
| ,6891 | ,585 | ,256 |
| ,6978 | ,585 | ,244 |
| ,7077 | ,585 | ,232 |
| ,7136 | ,569 | ,232 |
| ,7188 | ,569 | ,220 |
| ,7272 | ,569 | ,207 |
| ,7396 | ,569 | ,195 |
| ,7694 | ,569 | ,183 |
| ,7987 | ,569 | ,171 |
| ,8122 | ,554 | ,171 |
| ,8238 | ,538 | ,171 |
| ,8337 | ,538 | ,159 |
| ,8447 | ,538 | ,146 |
| ,8591 | ,538 | ,134 |
| ,8721 | ,538 | ,122 |
| ,8757 | ,523 | ,122 |
| ,8769 | ,523 | ,110 |
| ,8790 | ,508 | ,110 |
| ,8894 | ,492 | ,110 |
| ,9005 | ,477 | ,110 |
| ,9103 | ,477 | ,098 |
| ,9235 | ,477 | ,085 |
| ,9413 | ,462 | ,085 |
| ,9666 | ,446 | ,085 |
| ,9931 | ,431 | ,085 |
| 1,0179 | ,415 | ,085 |
| 1,0322 | ,400 | ,085 |
| 1,0385 | ,385 | ,085 |
| 1,0474 | ,369 | ,085 |
| 1,0596 | ,354 | ,085 |
| 1,0680 | ,338 | ,085 |
| 1,0834 | ,323 | ,085 |
| 1,0999 | ,308 | ,085 |
| 1,1067 | ,292 | ,085 |
| 1,1122 | ,292 | ,073 |
| 1,1144 | ,292 | ,061 |
| 1,1233 | ,292 | ,049 |
| 1,1357 | ,277 | ,049 |
| 1,1453 | ,262 | ,049 |
| 1,1601 | ,262 | ,037 |
| 1,1766 | ,262 | ,024 |
| 1,1914 | ,246 | ,024 |
| 1,2088 | ,231 | ,024 |
| 1,2355 | ,215 | ,024 |
| 1,2546 | ,200 | ,024 |
| 1,2667 | ,185 | ,024 |
| 1,2794 | ,169 | ,024 |
| 1,2909 | ,154 | ,024 |
| 1,3132 | ,138 | ,024 |
| 1,3456 | ,123 | ,024 |
| 1,3706 | ,108 | ,024 |
| 1,3894 | ,092 | ,024 |
| 1,4051 | ,077 | ,024 |
| 1,4169 | ,077 | ,012 |
| 1,4460 | ,062 | ,012 |
| 1,4960 | ,046 | ,012 |
| 1,5622 | ,031 | ,012 |
| 1,6088 | ,031 | ,000 |
| 1,7698 | ,015 | ,000 |
| 2,9217 | ,000 | ,000 |
